# Editorial: Microbial biofilms interacting with host mucosal surfaces

**DOI:** 10.3389/fcimb.2022.1049347

**Published:** 2022-10-07

**Authors:** Jean-Paul Motta, Anders P. Hakansson, Samuel A. Lee

**Affiliations:** ^1^ Institute of Digestive Health Research, INSERM U1220, Toulouse, France; ^2^ Division of Experimental Infection Medicine, Department of Translational Medicine, Lund University, Malmö, Sweden; ^3^ White River Junction Veterans Affairs (VA) Medical Center, White River Junction, VT, United States; ^4^ Section of Infectious Diseases and International Health, Geisel School of Medicine at Dartmouth, Hanover, NH, United States

**Keywords:** biofilms, mucosal surface, polymicrobial, host response, models, pathobionts

## Mucosal biofilms

Biofilms, a microbial community lifestyle, are ubiquitous within eukaryotic habitats (Flemming and Wuerts, 2019). Indeed, various mucosal surfaces such as the oral cavity (Bowen et al., 2018), the gastrointestinal tract (Motta et al., 2021), the skin (Ring et al., 2017; Bay et al., 2020), the vagina (Carson et al., 2021) and the nasopharynx (Marks et al., 2012) and lungs (Bjarnsholt et al., 2009) are naturally colonized by microbial biofilms, both in health and in disease. That said, it is becoming increasingly clear that we need to surpass our mental perception of what is a biofilm interacting with a mucosal surface, and more importantly how we should consider these microbial communities above traditional *in vitro* model systems (Bjarnsholt et al., 2021; Sauer et al., 2022). Interacting with mucosal surfaces, *in vivo* biofilms can be seen as extremely complex microbial ecosystems forming an ecological network, embedded in a self-produced and host biopolymer matrix, and either firmly, loosely or unattached to mucosal surfaces (Flemming and Wuerts, 2019; Sauer et al., 2022). Hence, understanding precisely how microbial biofilm interacts with mucosal surfaces, and how these interactions contribute to pathophysiology, bear significant interest for the whole scientific and medical community.

## Featured articles in this Research Topic

In the review paper by Guerra et al., a specific focus has been made on *Klebsiella pneumoniae*, a common inhabitant of various mucosal surfaces (lungs, skin, urogenital and gastrointestinal tract). It explores fundamental knowledge regarding biofilm biology of this species, and the threat of multidrug resistance of these biofilms is largely discussed. Biofilm phenotype still remain incompletely understood in this behavior, but is definitely worth investigating. Interestingly, a discussion was incorporated on the multispecies nature of naturally biofilm forming *Klebsiella* biofilms. It is noteworthy that transkingdom interaction with Candida may occur naturally in the oral mucosa and on the skin surface (particularly relevant for wound biofilms), but also in the gastrointestinal tract. The authors also explore different model systems to study *Klebsiella* biofilm formation (from static to dynamic; and *in vitro* to *in vivo* Galleria models). Finally, the authors discuss how some of these molecules could be used in future vaccines against this bacterium. Another review paper we have selected is a work from Weeks et al., focused on Non-typeable *Haemophilus influenzae* (NTHi), which is a puzzling pathobiont microorganism involved in lung infection in Chronic Obstructive Pulmonary Disease (COPD). In this paper, the authors summarize literature evidence of how NTHi adopts the biofilm lifestyle in the lung mucosal surface, with the activation of autoinducer-mediated quorum sensing systems, promotion of epithelial- and mucus-binding adhesins, as well as the genetic evidence for self-production of surrounding matrix. Interestingly, this paper also touches on the polymicrobial nature of NTHi biofilm *in vivo*, with many open up for novel and relevant research questions in the future. Importantly, in this review, the authors also discuss the clinical relevance of the biofilm phenotype in the particular context of COPD. Although these 2 review papers mostly focus on lung mucosal surfaces, several conceptual discussions may well be relevant for other mucosal surfaces, such as the skin, the gastrointestinal and urogenital tract.

Microbial endocrinology is an exciting emerging field, aiming for instance to understand the impact of host factors (e.g. hormones) during infection progression on mucosal surfaces. In that context, sexual dimorphism in bacterial infections associated with biofilm formation in cystic fibrosis (CF) are well-known, however there is much complexity beyond this statement. In the original paper from Al-Zawity et al., the authors isolated several clinical isolates of *Pseudomonas aeruginosa* in sputum of patients. They showed that estrogen promoted a biofilm phenotype in their *in vitro* static model, with various degree of effect on the physico-chemical architecture of biofilm matrices. Interestingly, no such effect using traditional laboratory strains was observed (a finding that may explain opposite findings from the literature). This could explain why *P. aeruginosa* found in CF patients undergoes more frequent mucoid conversion in female compared to male patients (Chotirmall et al., 2012). Here, the authors propose that estrogen modulates quorum sensing signaling in some, but not all, CF isolates. Overall, this interesting study justify the need to perform research on clinical strains, in addition to conventional laboratory models. It is noteworthy that the *in vitro* model used in this study is very simplified compared with *in vivo* settings in lung airways and future validation in a biologically relevant model would be highly appreciated in the field.

Another original study here, by Alves-Barroco et al., focused on *Streptococcus dysgalactiae* subsp. *dysgalactiae* (SDSD). This strain is a bovine mastitis-associated strain, but also implicated in human health. The paper adds experimental evidence to the field suggesting that SDSD strains can efficiently infect and form biofilms on a surface of human keratinocyte cells. Importantly, this paper uses an SDSD biofilm model in mice (subcutaneous catheter implantation), a model relevant for prosthesis-associated biofilm infection. Overall, this paper is a good example of how host mucosal factors can directly influence the transcriptome of mucosal biofilm *in vivo*, with upregulation of genes likely to be important for host colonization and survival there (including genes involved in the adhesion to host fibronectin). That said, future studies, such as those utilizing gene-specific genetic mutants, are needed to fully understand how this modified transcriptome on mucosal surfaces may contribute to pathogenic behavior. Finally, these findings may have a broader repercussion, as strains of a phylogenetically related species (*Streptococcus gallotycus*) are associated with colorectal cancer in humans (Aymeric et al., 2018), which is a disease associated with abnormal biofilm phenotype on the colon surface (Dejea et al., 2014; Motta et al., 2021).

Finally, a last original article in this Research Topic came from Wirth et al., who studied microbial biofilms interacting with the oral mucosa. Specifically, this paper explored in depth taxonomic diversity of oral biofilms associated with periodontitis in humans. What makes this study original, was that samples were not only taken from the periodontal pockets, but the saliva was also collected, from the same patients. Overall, authors revealed a change in taxonomic representations of healthy versus disease-associated biofilms (specifically with a drop in diversity). Although this study revealed that saliva may not exhaustively reflect taxonomy of the oral periodontal biofilms, it was still considered an efficient fluid to identify various microbiological biomarkers indicative of the oral health status. For instance, this paper demonstrated that most abundant periodontal pathogens of the subgingival biofilms were also present in the saliva. As a perspective, it remains to be clarified whether dispersal of the oral biofilm may be altered in disease situation and how this could influence the release of oral pathobionts reaching the gastrointestinal tract and causing disease there (Kitamoto et al., 2020).

## Conclusion and perspectives

In conclusion, this Research Topic gathered several papers that elucidate how mucosal biofilms may form and interact with the host mucosal surface. Studies presented here aimed at surpassing the classical views of biofilms being necessarily negative but rely on the fine alterations in biofilm features, and how they behave after exposure to host factors (e.g. hormones) that may be associated with disease initiation/perpetuation. As revealed in Al-Zawity et al. and discussed in Guerra et al., biologically relevant and *in vivo* biofilms models are sorely needed to provide convincing evidence of the clinical relevance of the findings presented. Additionally, the specific interactions within polymicrobial, including cross-kingdom, biofilms and their impact on pathogenesis remains an area needing further study. Further development of clinically significant *in vivo* polymicrobial biofilm models will be of great value in this effort. Although mucosal biofilms are central in the pathophysiology of chronic disorders, they can also contribute to homeostatic development of many physiological functions. Hence, as a perspective on works presented here, additional works are now needed to understand how commensal and/or probiotic biofilms could contribute to health benefits in mucosal surfaces.

## Author contributions

J-PM was a guest associate editor of this Research Topic and wrote this editorial. AH was a guest associate editor, acted as an editor for one paper and as an author for a review paper in this Research Topic, and contributed to this editorial. SL was a guest associate editor of this Research Topic, acted as an editor for one paper in this Research Topic, and contributed to this editorial. All authors contributed to the article and approved the submitted version.

## Acknowledgments

We deeply thank all the authors and reviewers who have participated in this Research Topic.

## Conflict of interest

The authors declare that the research was conducted in the absence of any commercial or financial relationships that could be construed as a potential conflict of interest.

## Publisher’s note

All claims expressed in this article are solely those of the authors and do not necessarily represent those of their affiliated organizations, or those of the publisher, the editors and the reviewers. Any product that may be evaluated in this article, or claim that may be made by its manufacturer, is not guaranteed or endorsed by the publisher.
